# The effect of a mystery shopper scheme on prescribing behavior in primary care: Results from a field experiment

**DOI:** 10.1186/s13561-020-00290-z

**Published:** 2020-09-24

**Authors:** Roland Cheo, Ge Ge, Geir Godager, Rugang Liu, Jian Wang, Qiqi Wang

**Affiliations:** 1 Center for Economic Research, Shandong University, 27 Shanda Nanlu, Jinan, Shandong, 250100 P.R. China; 2 Department of Health Management and Health Economics, University of Oslo, P.O. Box 1089 Blindern, Oslo, 0317 Norway; 3 Health Services Research Unit, Akershus University Hospital, Sykehusveien 25, Nordbyhagen, 1478 Norway; 4 School of Health Policy & Management, Nanjing Medical University, 101 Longmian Avenue, Jiangning District, Nanjing, 211166 P.R. China; 5 Center for Global Health, Nanjing Medical University, 101 Longmian Avenue, Jiangning District, Nanjing, 211166 P.R. China; 6 Dong Fureng Institute of Economic and Social Development, Wuhan University, 54 Lishi Hutong, Dongcheng District, Beijing, 100010 China; 7 Center for Health Economics and Management in School of Economics and Management, Wuhan University, 299 Bayi Road Wuchang District, Wuhan, 430072 China; 8 School of Economics, Xi’an University of Finance and Economics, 360 Changning Avenue, Chang’an District, Xi’an Shanxi, 710100 China

**Keywords:** Field experiment, Primary care, Prescription, Information and product quality, Social responsibility, C93, I11, I18, L15, M14

## Abstract

**Background:**

Health care systems in many countries are characterized by limited availability of provider performance data that can be used to design and implement welfare improving reforms in the health sector. We question whether a simple mystery shopper scheme can be an effective measure to improve primary care quality in such settings.

**Methods:**

Using a randomized treatment-control design, we conducted a field experiment in primary care clinics in a Chinese city. We investigate whether informing physicians of a forthcoming mystery shopper audit influences their prescribing behavior. The intervention effects are estimated using conditional fixed-effects logistic regression. The estimated coefficients are interpreted as marginal utilities in a choice model.

**Results:**

Our findings suggest that the mystery shopper intervention reduced the probability of prescribing overall. Moreover, the intervention had heterogeneous effects on different types of drugs.

**Conclusions:**

This study provides new evidence suggesting that announced performance auditing of primary care providers could directly affect physician behavior even when it is not combined with pay-for-performance, or measures such as reminders, feedback or educational interventions.

## Background

As noted by Arrow [1], asymmetric information about product quality is a fundamental characteristic of the medical care market. The providers of health services are experts who typically hold information that is superior to that of the patients and the payers of the services. When the presence of asymmetric information limits provider quality assurance, it affects the providers’ incentive for quality delivery. Recent health reforms in many countries are designed to encourage quality improvements by linking financial incentives to observable indicators of quality. When feasible, policymakers often take advantage of advances in information and communication technology in developing of policy measures, such as by designing mechanisms for provider payment based on routinely collected data on provider activity and performance. The Quality and Outcomes Framework (QOF) in the United Kingdom is an example of an extensive pay-for-performance program that relies on advanced infrastructure in the form of health registers and patient lists when measuring provider performance.

Many health care systems are still characterized by limited availability of provider performance data and patient registers. Without routinely collected performance data, the implementation of an advanced pay-for-performance system is not feasible in all countries. In the presence of asymmetric information on service quality, the degree of asymmetry can be influenced by introducing simple auditing schemes that do not rely on routinely collected register data on every provider. Such performance auditing is often designed to improve the quality of services by evaluating the quality against standards and can be implemented without necessarily linking financial incentives to performance. As described by Dranove [2], health plans and hospitals frequently contribute to quality assurance mechanisms by collecting and voluntarily disclosing quality information. While knowledge of hospital performance is a necessity in modern hospital management, auditing primary care physicians more likely requires an external initiative. As reviewed by Ivers and Oxman [3], most intervention studies on auditing focus on the effect of auditing when combined with other measures, such as *reminders* [4, 5], *feedback* [5–11] or *educational interventions* [12, 13]. In a recent study by Östervall [14], however, the effect of auditing primary care physicians’ practice in Sweden is separated from the effect of reminding physicians and patients about the inappropriate use of antibiotics. The reminders are found to have a substantial effect on prescribing, whereas introducing audits does not significantly influence physician prescribing behavior. Our study relates to the study by [14] in that we aim to quantify the effect of announced auditing on prescribing behavior.

We question whether announced auditing in the form of a mystery shopper scheme can be an effective measure to improve health care quality in primary care markets where routinely collected performance data is not available, and we propose to identify this effect by applying the method of mystery shopping in a randomized treatment-control design. Mystery shopping is frequently used for performance measurement to reduce the asymmetry of information in industries organized as chains. Mystery shoppers interact with product or service providers following specific scripts of tasks and report back detailed information on the experience. A mystery shopper scheme thus enables decision makers to acquire performance information on subdivisions of an organization, which can be used for pure monitoring purposes as well as performance-based payment [15]. Mystery shopper schemes can be customized to suit different purposes, and using mystery shoppers to collect information for research purposes has become more common in recent years. The key element of a mystery shopper is that parties that are audited are not informed about the mystery shopper’s identity and when audits will occur. Decades ago, the mystery shopping approach was adopted in the health domain to study provider behavior, and it has been proved valuable to society [16]. In a health context, mystery shoppers are commonly referred to as *pseudopatients*, *simulated patients*, *standardized patients* or *surrogate patients*. Using pseudopatients involves an element of deception, which generally involves careful ethical considerations, especially in the health research domain. Application of this method can be ethically justified, however, as long as individuals’ confidentiality is protected, risks to the research subjects are minimal and the research is potentially valuable in furthering our knowledge on the subject [17]. This project was subject to ethical assessment and was approved by the Data Protection Official for Privacy in Research, Norwegian Social Science Data Services, which serves as the institutional review board for the University of Oslo.^1^

The quality measure applied in our study is the physician’s prescribing behavior when the patient presents a specific set of symptoms. The symptoms presented by the pseudopatients in this study are symptoms of a mild common cold. As reviewed by Simasek and Blandino [18] and Allan and Arroll [19], medical studies on various treatments for the common cold do not show clear benefits, and adverse side effects from inappropriate treatment can potentially harm patients. In addition, financial costs paid by patients when purchasing medications contribute negatively to patients’ overall welfare. Hence, whether or not medication is prescribed is an observable and convenient quality measure in our specific study setting. In general, prescribing behavior in primary care is a highly relevant quality aspect, as inappropriate prescribing of medication has become a global public health challenge. According to the World Health Organization [20], more than half of medical prescriptions worldwide are inappropriate, causing not only adverse health outcomes but also increasing health expenditures. A typical example is the overprescribing of antibiotics. This practice is common in many countries, leading to widespread resistance against medications used for treatable bacterial infections [21–24]. Governments are increasingly implementing guidelines and regulations to curb such misuse of medications. The literature reveals, however, that antibiotics are prescribed too often, even in the presence of guidelines and gatekeeping [25–27].

We conducted a field experiment on physicians from small private clinics in Jinan, China. The majority of the physicians in our sample are owners or co-owners of the clinics. The profit from medication sales is often their main source of income, as they most often do not charge consultation fees. We randomized clinics into either a treatment or control group. We applied a similar audit methodology and script as Currie et al. [26, 27] and announced a forthcoming mystery shopper audit only to clinics in the treatment group. Physicians’ prescribing behavior was categorized into four types, corresponding to the inclusion of antibiotics, other prescription drugs (Other Rx), over-the-counter drugs (OTC), and alternative and nonpharmacological treatments (Alternatives) in the prescription. We found that the mystery shopper intervention unambiguously reduced the mean marginal utility of prescribing drugs and thereby the probability of prescribing overall. Moreover, the average reduction in prescribing was mostly driven by reductions in Other Rx and OTC.

This paper contributes to the literature using field experiments to acquire knowledge on key mechanisms in health service delivery. To our knowledge, this is the first paper to examine whether providers change behavior in response to preannouncement of a mystery shopper audit. In addition to this innovation, a strength of the paper is the use of a randomized treatment-control design to identify the intervention effect. This paper provides new evidence suggesting that auditing primary care providers can directly affect physician behavior, even when it is not combined with pay-for-performance, or other measures such as reminders, feedback or educational interventions.

### Theoretical background and hypotheses

The patient-physician relationship is commonly described as a case of (imperfect) agency [28]. The patient (principal) consults the physician (agent), who is an expert with superior information regarding health and expected treatment effects.^2^ Under perfect physician agency, the optimal treatment for the patient will coincide with the optimal treatment option for the physician. In our study setting, income from selling medications comprises a substantial share of physicians’ income. Financial incentives to prescribe drugs result in conflicting objectives between patients and physicians, as it becomes costly to always behave as a perfect agent on behalf of the patient.

We studied the case of a patient with a common cold, where prescribed medication is not expected to contribute to positive health benefits. When the patient needs to pay out-of-pocket for medication, one may argue that a rational patient would refrain from drug purchase if the patient and physician were equally well informed. Upon seeing a patient with minor symptoms of a common cold, the physician decides whether or not to prescribe medication.

We assume that the patient passively accepts the physician’s treatment recommendation and indicate the prescribing choice by *a*, where *a*=1 if the physician chooses to prescribe, and *a*=0 otherwise. We assume that the physician’s net profit, *π*, from prescribing is positive. The physician’s choice affects patient’s net benefit, *V*(*a*), defined by health benefit measured in money minus cost of medication. In the case of the common cold, prescribing reduces the patient’s net benefit, *V*(1)<*V*(0), since prescribed medication is not expected to provide positive health benefits, and the patient incurs costs.

We assume that physicians are partly altruistic, and, similar to Farley [29], we include the physician’s concern for the patient’s overall well-being when specifying the physician’s objective. When the physicians are informed of a forthcoming mystery shopper audit, it implies that their service quality and professionalism can be acknowledged by a relevant institution. We propose that the alternative *not prescribe*, being medically appropriate and beneficial to the patient while yielding low physician profit, can become more rewarding after receiving information of a forthcoming mystery shopper audit: In the presence of a mystery shopper scheme, information on medical decisions will reach a broader audience than what is the case in a conventional physician-patient encounter. As described by Bénabou and Tirole [30], the physician’s objective might include other elements, such as “recognition by others” or “social stigma” in conjunction with profit motive and concern for patients, and therefore, they may behave differently when a mystery shopper scheme is introduced.

We indicate the existence of a mystery shopper scheme by *T*, where *T*=1 when a mystery shopper scheme exists and *T*=0 otherwise. The element of “recognition by others” or “social stigma” can be included additively in the physician objective as a function *S*(*a*;*T*), which introduces a stigma effect from prescribing in the context of a mystery shopper scheme. We assume that in the absence of a mystery shopper scheme (*T*=0), stigma does not affect the provider objective, i.e., *S*(1;0)=*S*(0;0). In the case of mystery shopping (*T*=1), however, prescribing unnecessary medication results in a negative stigma effect: *S*(1;1)<*S*(0;1). The objective for a physician who cares about social stigma besides profit and patients’ net benefit can be expressed as:

1 $$ U(a;T)=\pi a+b V(a)+ c S(a;T)  $$

where the preference parameter, *b*>0, indicates the weight the physician attaches to the patient’s net benefit, and *c*≥0 indicates the preference weight of social stigma in the physician’s objective function. We assume that physicians behave as if they are maximizing (1).

In the absence of a mystery shopper scheme (*T*=0) where *S*(1;0)=*S*(0;0), a physician would prescribe if *U*(1;0)>*U*(0;0), where *U*(1;0)=*π*+*bV*(1)+*cS*(1;0) and *U*(0;0)=*bV*(0)+*cS*(0;0). Under the assumption that physicians maximize (1), physicians with low altruism, $b < \frac {\pi }{V(0)-V(1)}$, will prescribe; those with a high altruism, $b > \frac {\pi }{V(0)-V(1)}$, will not prescribe; and physicians with $b = \frac {\pi }{V(0)-V(1)}$ will be indifferent to prescribing choices. In the case of preference heterogeneity in the population of physicians, preference variation will cause practice variations in terms of heterogeneous prescribing choice for a given patient.

In the presence of a mystery shopper scheme (*T*=1), a physician’s decision depends on the sign of *U*(1;1)−*U*(0;1), where *U*(1;1)=*π*+*bV*(1)+*cS*(1;1) and *U*(0;1)=*bV*(0)+*cS*(0;1). It can be shown that in a population of physicians that maximize (1) with varying *b*, introducing a mystery shopping scheme will cause a change in behavior for a subset of physicians.

**Fig. 1:**
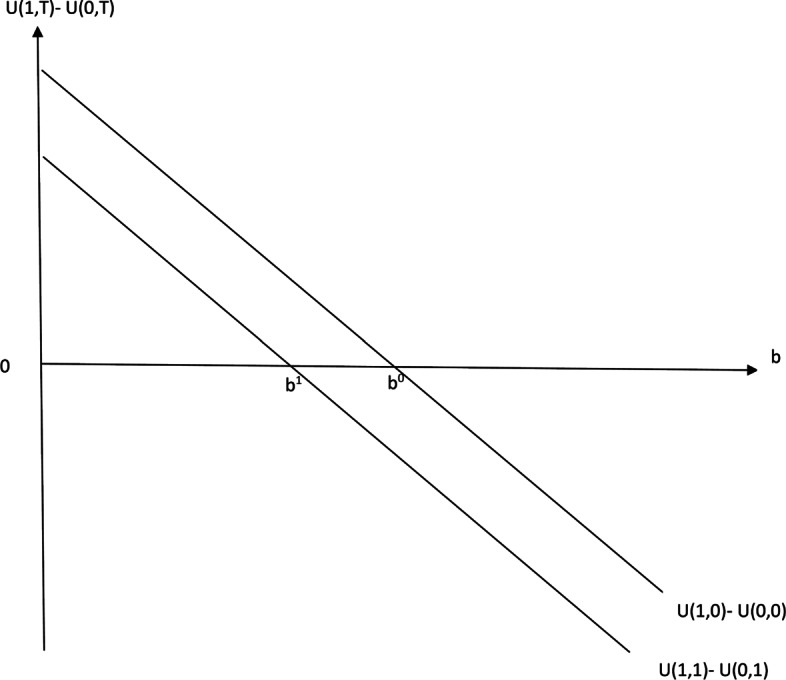
Incremental utility from prescribing, with and without mystery shopping

The result can be illustrated by studying the optimal choice for the physician who is indifferent to prescribing in the absence of mystery shopping, with the altruism parameter given by $b^{0} = \frac {\pi }{V(0)-V(1)}$. Introduction of a mystery shopper scheme will cause this physician to strictly prefer the alternative *not prescribe*, since *U*(1;1)−*U*(0;1)=*c*(*S*(1;1)−*S*(0;1))<0. The result is illustrated in Fig. 1. The two lines represent incremental utility from prescribing, with and without a mystery shopper scheme. Under the assumption that physicians maximize (1), physicians choose *prescribe* whenever *U*(1;*T*)−*U*(0;*T*)>0 and *not prescribe* whenever *U*(1;*T*)−*U*(0;*T*)<0. We see that in the absence of mystery shopping, the physician’s incremental utility from choosing to prescribe is negative for physicians with *b*>*b*^0^. Introducing mystery shopping shifts the incremental utility curve downwards, and now indifference in the prescribing decision occurs for a lower level of altruism *b*=*b*^1^, implying that a mystery shopper scheme will cause a change in behavior for a subset of physicians with altruism parameters *b*∈(*b*^1^,*b*^0^).

Based on the model results, we specify our main hypothesis:

**The probability of physicians prescribing medication to patients with symptoms of a minor common cold will be reduced by announcing a mystery shopper scheme.**

A plausible extension of the model is to allow for heterogeneous stigma effects over different types of prescribed medications. Therefore, a secondary hypothesis can be specified:

**The effects of announcing a mystery shopper scheme are heterogeneous over different types of prescribed medications.**

We test our hypotheses in a setting where primary care physicians earn a net profit from selling their prescribed drugs and the patients pay the full price out-of-pocket.

## Methods

### Experimental design and procedure

The literature reveals that Chinese physicians prescribe medication, especially antibiotics, when they should not [25–27]. An important cause of medication overprescribing in China is the financial incentives. Revenues from selling medication have become more important to hospitals since the early 1980s, when the government began to reduce financial support to hospitals [31]. For physicians in private clinics, profit from medication sales is often the main source of income, as they most often do not charge consultation fees. To mitigate incentives for overprescribing in China, various reforms have been implemented by the Chinese government since 2009. In general, most of the regulation and reforms target private and public hospitals rather than private clinics. In 2010, the Health Ministry separated doctors’ pay from prescription drug sales to curb the widespread prescription of antibiotics in hospitals [32]. In 2011, the Health Ministry also regulated antibiotic prescription for hospitalized patients and outpatients and set targets at less than 60% and 20% of all prescriptions. In addition, antibiotic utilization in hospitalized patients were set at less than 40 daily defined doses per 100 patient days [33]. However, these reforms have not proven effective [34]. We conducted a randomized field experiment in private clinics in China to investigate if preannouncement of a mystery shopper audit could improve the quality of primary health care services.

***Sample and randomization***

Our field experiment was performed in Jinan, the capital city of Shandong province in China. By performing the experiment among small walk-in private clinics where no patient ID is required and no patient records are kept, we could randomly assign pseudopatients to clinic visits. It might be more challenging to conduct a similar field experiment in a country where durable physician-patient relations, often formalized as patient list systems, are common. We received support from the School of Public Health at Shandong University and Qilu Health Service Center, which is affiliated with the largest public hospital in Jinan (Qilu Hospital); and this support added substantial credibility to the mystery shopper intervention.

From official Chinese registers in the Health and Family Planning Commission of Jinan Municipality, we identified 118 primary care clinics in Jinan based on these criteria: the clinic is for-profit with only one practicing physician, is located within the five districts of Jinan city,^3^ has a valid license on the date of the experiment, and provides general medicine.^4^ From the list of suitable clinics, we then randomly assigned 48 clinics to the control group, 48 clinics to the treatment group, and the remaining 22 clinics served as backups. In case any visited clinic was permanently closed, one random clinic from the 22 backups could replace the closed one. According to our prior information on prescribing in primary care, we expected that medications would be prescribed in a majority of consultations. We aimed to assess whether the intervention could generate a substantial reduction in inappropriate prescribing. Our sample size was based on power calculations. With a sample size of 96, the likelihood of correctly rejecting the null-hypothesis (the intervention has no effect) in a Pearson’s *χ*^2^ test, given an effect size of 30 percentage points, is 80% when significance level is set at the conventional level of 5%.

***Mystery shopper audit***

Following Moriarty et al. [35] and Bisgaier and Rhodes [36], we carried out two mystery shopper audits on all 96 clinics in November and December 2015. A time-line of the field experiment is provided in Table 1. Throughout the first audit, we collected baseline data on the characteristics of the clinics and the practicing physicians and their prescribing behavior. Based on the second audit, we compared differences in prescribing behavior between the treatment and control groups.

**Table 1:** Timeline of the field experiment

	**Dates**
**First audit**	30th November, 1st December and 2nd December 2015
**Intervention**	7th December, 8th December and 9th December 2015
**Second audit**	28th December, 29th December and 30th December 2015

In both audits, pseudopatients presented symptoms of the common cold to the physician according to a script (see Appendix C) and a protocol (see Appendix D). They described their symptoms as “feel fatigued, have a low grade fever, slight dizziness, a sore throat and a poor appetite”, and they told the physician that their body temperature was 37 ^∘^C in the morning. The pseudopatients were explicitly instructed not to say to the physician that they have a cold. They allowed the physician to measure their temperature and/or visually inspect their throat. The pseudopatients were strictly instructed to refuse any other treatment or diagnostic test by the physician. If the physician prescribed any medication, the patient was instructed to memorize the names and the pharmaceutical companies of all the medications prescribed. The patient was then to ask for the price of the prescription. The budget for drug purchasing was set at 20 Yuan. The cost for a one-time clinic visit due to a mild common cold would typically be lower. It is important to note that this budget was never revealed to the physician, and the patient’s purchasing decision was announced after the drugs were prescribed. Hence, physicians’ prescribing behavior was measured by drugs prescribed, not drugs purchased.

A pseudopatient was always accompanied by a fellow student during the audits. The fellow students observed the number of additional patients in the waiting room, the number of additional physicians and patients in the office, the gender and age of the practicing physician and helped the pseudopatient memorize the medication names. The pseudopatient and the accompanying student completed a data collection sheet together after leaving the clinic.

***Mystery shopper intervention***

The intervention of announcing a forthcoming mystery shopper audit was conducted three weeks before the second audit. A representative of the research project visited the clinics in the treatment group one by one to announce the mystery shopper audit. The announcement was made in person by presenting a letter containing information about a current project at Shandong University (see Fig. 3 for an English translation of the project description letter in Appendix E). The project is about quality evaluation of primary care services in Jinan, particularly service, professionalism, and adequacy of treatment. The clinics were informed that an anonymous patient would visit the clinics and collect information about the treatment decision and then evaluate the quality of care. To enhance the credibility of the research project, we offered the clinics three ways to receive feedback from the quality assessment: publicly available feedback (results published on the Shandong University website), feedback in private (results only received by the clinic) or no feedback.^5^ The representative read the project description with the physician and ensured that the physician understood the project. In addition, Qilu Health Science Center, affiliated with Shandong University and one of the largest public hospitals (Qilu Hospital) in Jinan, provided an endorsement letter to support the project (see Fig. 4 for an English translation of the endorsement letter in Appendix E). The representative presented the endorsement letter to the physician and left both the stamped project description and the endorsement letter at the clinic.

***Training of the pseudopatients***

The audits were performed by 12 healthy pseudopatients, each accompanied by a fellow student, recruited from the School of Public Health, Shandong University.^6^ Each pseudopatient visited 8 clinics in both audits. Each pair of students (the pseudopatient and the accompanying student) underwent 10 hours of training in total on the 10th and 11th of October 2015. The purpose of the extensive training of the pseudopatients and the accompanying students was to ensure adherence to the script and protocol in order to reduce data variations due to subjective interpretations by the pseudopatients and to enhance the credibility of the pseudopatients so that the physicians are not able to identify them. On the first day, they went through a review of the types of antibiotics and cold medicines on the market. They also had to rehearse and role play using the script. At the end, they practiced filling out the information sheet. Training on the second day involved practice visits to clinics that were not in the 118 identified clinics. To further ensure adherence to the script, the data collection sheets and the physician-patient dialogues from the practice visits were discussed. The teams of pseudopatients were randomly assigned to clinics. They did not visit any clinic twice, and they were not informed about whether the clinics were in the treatment or control group.

### Ethical considerations

The mystery shopper audit has been used in the health care domain for decades and has been developed into a scientifically sound experimental method that provides unique and valuable knowledge to society in both developing and developed countries (see for example [16, 35, 53, 54]). The use of deception is controversial in science, and there is no unanimous classification across disciplines. The main ethical dilemma in our study is that the healthy pseudopatients provide incorrect information to the physician when describing their state of health. However, following the ethical analysis of Rhodes and Miller [17], it can be ethically justified as long as confidentiality of research subjects is ensured, risks to the research subjects are minimal and the research is potentially valuable to human knowledge.

To ensure the safety of the pseudopatients, they were always accompanied by a fellow student, so a team of two students always traveled together. Furthermore, the pseudopatients, being students of the School of Public Health, had at least one semester of basic medical training and were specifically instructed to refuse any treatment and/or diagnostic test by the physician except for temperature measuring and visual inspection of the throat. To protect the physicians’/clinics’ privacy, we generated a unique series of ID numbers identifying each clinic. The sheet of paper linking ID numbers with clinic addresses was destroyed after the visits, so data from the clinics could not be traced to a particular clinic or physician, even by the researchers. In addition, the field experiment also contributed positively to the revenues of the clinics in the study sample, since physicians gained profit by selling prescribed medications.

### Data

**Fig. 2:**
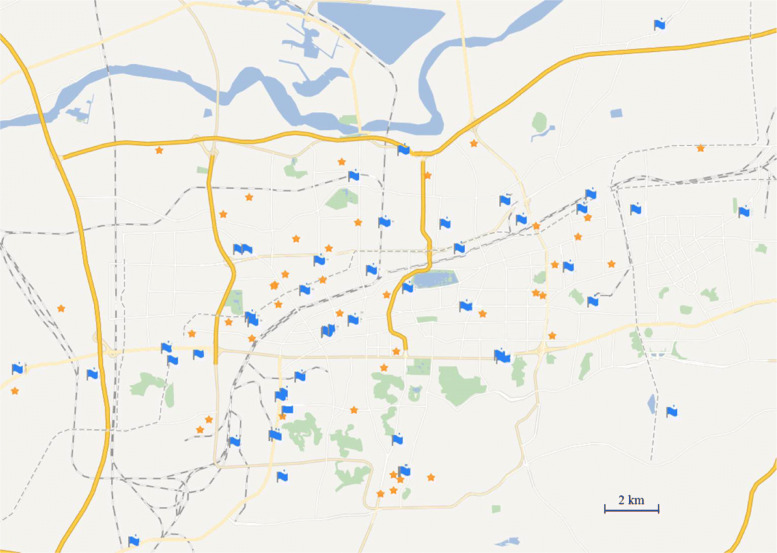
Map of locations of sampled 96 clinics Stars: the control group; Flags: the treatment group

The 96 clinics were randomized into the treatment and control groups. The map (see Fig. 2) indicating the locations of the clinics in the treatment and control groups provides a rough impression that the treatment and control clinics were randomly scattered throughout Jinan city. Table 2 reports the inclusion of treatment and control clinics over the five districts in the city. There is no significant difference in representation of treatment and control clinics over the districts (*p*-value =0.359,*χ*^2^ test).

**Table 2:** Locations of sampled 96 clinics

	**District 1**	**District 2**	**District 3**	**District 4**	**District 5**	**Total**
**Control**	10	11	3	12	12	48
**Treatment**	12	5	7	14	10	48

Table 3 presents summary statistics from our sample at the clinic level. We collected data on the size of the clinics, measured by the number of additional physicians and patients^7^ in a physician’s office, and the number of additional patients in the waiting room. Based on the results from Mann-Whitney-Wilcoxon (MWW) tests, there are no significant differences in observed characteristics between clinics in the treatment and control groups in either audit. In the second audit, one clinic in the control group had become a drug store, and one clinic in the treatment group was closed. Therefore, we removed these two clinics from our sample, and data from 94 clinics was used in our study of the second audit. During the experiment, it was discovered that many of the clinics registered as a single-physician unit had more than one physician employed. Due to the design and confidentiality of individual physicians, we cannot ensure a one-to-one match between physicians in the first and second audit.

**Table 3:** Clinic characteristics

	**Control**			**Treatment**			**MWW**
**Variables**	**Mean**	**Sd.**	**N**	**Mean**	**Sd.**	**N**	***p*-value**
**Audit 1**							
# of additional physicians in the office	0.333	0.808	48	0.354	0.758	48	0.792
# of additional patients in physician’s office	0.979	1.436	48	0.938	1.359	48	0.865
# of additional patients in the waiting room	0.250	0.636	48	0.375	0.672	48	0.182
**Audit 2**							
# of additional physicians in the office	0.617	1.054	47	0.447	0.880	47	0.451
# of additional patients in physician’s office	1.191	1.313	47	1.511	1.932	47	0.653
# of additional patients in the waiting room	0.234	0.560	47	0.213	0.508	47	0.826

Table 4 describes the characteristics of the practicing physicians for both audits. We can see that the number of male and female physicians in the control and treatment groups are similar for the first audit, while there are more females in the control and more males in the treatment group in audit two. Physicians’ age was observed and categorized into four groups. Physicians’ characteristics could potentially influence their prescribing patterns, and the intervention might therefore have varying impacts on physicians of different genders and ages. For this reason, we control for physician characteristics when we analyze the intervention effect on physician prescribing behavior.

**Table 4:** Physician characteristics

		**Audit 1**				**Audit 2**			
		**Control**		**Treatment**		**Control**		**Treatment**	
**Variables**		**Freq.**	**N**	**Freq.**	**N**	**Freq.**	**N**	**Freq.**	**N**
Gender	Male	24	48	23	48	19	47	25	47
	Female	24	48	25	48	28	47	22	47
Age	≤30	2	48	2	48	2	47	2	47
	[31,40]	24	48	26	48	16	47	26	47
	[41,50]	12	48	18	48	21	47	12	47
	≥51	10	48	2	48	8	47	7	47

Age is categorized into four levels: younger than or equal to 30 years old; between 31 to 40 years old; between 41 to 50 years old; older than or equal to 51 years old

Based on the prescribing data and reviews of treatments for the common cold [18, 19], we categorize physicians’ prescribing into four prescription types: antibiotics, other prescription drugs (Other Rx), over-the-counter drugs (OTC), and alternative and nonpharmacological treatments (Alternatives). Despite the fact that the common cold is a viral illness, antibiotics are frequently prescribed for this illness in China. Medical studies have recorded evidence that antibiotics provide no benefit and can potentially cause harm by increasing bacterial resistance [37, 38]. Other prescription drugs are not recommended when only mild cold symptoms are presented due to risks of adverse effects and unclear benefits, especially in our experiment where no other diagnostic tests were ordered other than a visual inspection of the patient’s throat and a measurement of the temperature. While there is no cure for the common cold, most over-the-counter drugs are directed at relieving certain symptoms. Examples are paracetamol (acetaminophen), ibuprofen or other pain relievers for body aches or a headache and decongestant nasal sprays. Considering the side effects, they are in general not recommended given the absence of symptoms. In general, OTCs include a wide range of medicines of which the benefits are unclear but likely small in adults. Alternative and nonpharmacological treatments include, for example, vitamin C supplements and cough drops, and the benefits are likely absent [19]. In addition to unclear health benefits, prescriptions for any medications increase patients’ financial costs.

Table 5 summarizes physicians’ prescribing behavior from the control and treatment groups in both audits. The large majority of physicians prescribed at least one type of drug to the patients in both audits. In the second audit, where all the physicians in the control group provided some medication to the pseudopatients, significantly fewer physicians (*χ*^2^ test *p*-value=0.022) in the treatment group (89.4%) provided medication. There were only a few physicians who did not prescribe any drug at all: 3 in the control group and 6 in the treatment group in the first audit, and 0 in the control group and 5 in the treatment group in the second audit. From the first audit data, we can see that OTCs were the most prescribed alternative, with more than 80% of physicians choosing to prescribe them. OTCs were followed by antibiotics, which were prescribed by around two-thirds of physicians. This observation clearly confirms the prevalence of antibiotic overprescribing in China in the case of the common cold and is similar as the previously reported rate in experimental studies [26]. The less commonly prescribed treatments were Other RX and Alternatives, provided by approximately 12% and 3% of physicians, respectively. From the second audit data, we observed higher prescription rates of antibiotics and lower rates of Other Rx, OTC and Alternatives in the treatment group compared to the control group. Overall, the qualitative prescribing pattern described by the ranking of prescribing rates of the four types of drugs is the same in both audits and for both groups.

**Table 5:** Physician prescribing behavior

	**Control**				**Treatment**			
**Variables**	**Mean**	**Sd.**	**Freq.**	**N**	**Mean**	**Sd.**	**Freq.**	**N**
**Audit 1**								
Prescribe any drug	93.8%	0.245	45	48	87.5%	0.334	42	48
Antibiotics	62.5%	0.489	30	48	66.7%	0.476	32	48
Other Rx	12.5%	0.334	6	48	12.5%	0.334	6	48
OTC	85.4%	0.357	41	48	81.3%	0.394	39	48
Alternatives	2.1%	0.144	1	48	4.2%	0.202	2	48
**Audit 2**								
Prescribe any drug	100%	0	47	47	89.4%	0.312	42	47
Antibiotics	57.4%	0.500	27	47	68.1%	0.471	32	47
Other Rx	23.4%	0.428	11	47	10.6%	0.312	5	47
OTC	85.1%	0.360	40	47	76.6%	0.428	36	47
Alternatives	8.5%	0.282	4	47	6.4%	0.247	3	47

Prescribe any drug: prescribe at least one type of drug; Other Rx: Other prescription drugs; OTC: Over-the-counter drugs; Alternatives: Alternative and nonpharmacological treatments

### Empirical strategy

The decision to prescribe a drug to a patient is a standard discrete economic choice [39], and the choice modelling literature comprises a rich toolbox for analyzing how individuals’ choice combinations are affected by the characteristics of the available alternatives, as well as differences in context [40]. Choice models are now commonly used in studies applying experimental data (see for example [11, 55–58]). We examine and quantify the intervention effect on prescribing choices of the individual physicians. The prescribing choice can, without loss of generality, be split into a sequence of choices, where the physician first decides whether or not to prescribe antibiotics, and then they decide whether or not to include other types of drugs, one by one, until a complete prescription is chosen.

We estimate the intervention effects using a standard conditional fixed-effects logit model, which allows us to quantify the observed heterogeneity of prescribing patterns across different categories of drugs with and without the intervention. The physician’s prescribing decision is indicated by *y*_*it*_, where we use the indices *i*=1,2,...,*N* for physician, and *t*=1,2,3,4 for the types of drug that physician *i* decides to include or exclude in the medical treatment of the patient. The physician’s prescribing decision for each drug type is a binary choice variable such that *y*_*it*_=1 if the physician prescribes drug *t*, and *y*_*it*_=0 otherwise. Let the mean marginal utility for physician *i* of prescribing drug *t* be denoted by $v_{it}^{\ast }$. We allow $v_{it}^{\ast }$ to depend on whether or not physician *i* is in the treatment group, by defining it as $v_{it}^{\ast }=v_{t}\left [1+\gamma _{t}I_{i}\right ]$, where *v*_*t*_ denotes the mean marginal utility of prescribing drug *t* for physicians without the intervention. The potential effects of the intervention are captured by the inclusion of the intervention dummy *I*_*i*_. The intervention effect *γ*_*t*_ is allowed to vary over the different types of drugs. In the special case where the intervention effects, *γ*_*t*_, are all zero, we have $v_{it}^{\ast }=v_{t}$ for physicians in both the treatment and control groups. Letting *α*_*it*_ be any unobservable heterogeneity that is fixed for physician *i* when deciding on whether to prescribe drug *t*, the conditional logit probability of physician *i* prescribing drug *t* is given by:

2 $$ Pr\left(y_{it}=1\right)=\frac{\text{exp}\left(\alpha_{it} +v_{it}^{\ast}\right) }{\text{exp}\left(\alpha_{it}\right) + \text{exp}\left(\alpha_{it} +v_{it}\ast\right)}  $$

From Eq. (2) and the definition of $v_{it}^{\ast }$, we see that when the intervention does not have any effect, i.e., *γ*_*t*_=0, we have $v_{it}^{\ast }=v_{t}$. This means that the marginal utility of prescribing drug *t*, and thus the probability of prescribing, do not differ between the treatment and the control groups. The *γ*_*t*_ parameter captures the causal effect of the intervention on the marginal utility of prescribing. When *γ*_*t*_ is positive (negative), the interpretation is that the probability that the physicians’ treatment recommendation includes drug *t* is positively (negatively) affected by the intervention.

A convenient feature of the conditional fixed-effects logit model is that the fixed effects *α*_*it*_ are conditioned out of the likelihood function, since Eq. (2) reduces to $\frac {\text {exp}\left (v_{it}^{\ast }\right) }{1 + \text {exp}\left (v_{it}^{\ast }\right)}$ [41]. By means of a conditional logit model, we may therefore acquire robust estimates of the mean marginal utilities without the intervention, *v*_*t*_, and the intervention effect, *γ*_*t*_. Extending from single item choices to choices of bundles is trivial, and a clear deduction is provided by Hole [42]. Applying a robust method that enables analysis of how the intervention affects both the probability of prescribing and the composition of the prescribed drugs is a key feature of the empirical analysis. We estimate the conditional fixed-effects logit models by means of the clogit module in Stata 16. The same models are applied to data from the first and second audit, respectively. The intervention effects presented in Table 6 are estimated using data from the second audit. In Appendix A, we present model estimates based on the first audit, providing evidence that physicians in the treatment and control groups did not behave significantly different prior to the intervention.

**Table 6:** Intervention effects on physician prescribing

	**Model 1**	**Model 2**
**Panel A: Prescribing pattern (*v***_***t***_**)**		
Antibiotics	0.630^***^	0.300
	(0.197)	(0.269)
Other Rx	-1.481^***^	-1.186^***^
	(0.294)	(0.367)
OTC	1.551^***^	1.743^***^
	(0.269)	(0.327)
Alternatives	-2.418^***^	-2.375^***^
	(0.531)	(0.688)
**Panel B: Average intervention effect (*γ*)**		
	-0.214^***^	
	(0.064)	
**Panel C: Heterogeneous intervention effects (*γ***_***t***_**)**		
Antibiotics		0.458
		(0.304)
Other Rx		-0.943^**^
		(0.434)
OTC		-0.557^**^
		(0.264)
Alternatives		-0.311
		(0.519)
Number of observations	752	752
Log-Likelihood	-175.4	-173.2
Pseudo *R*^2^	0.327	0.336
AIC	360.9	362.4
BIC	384.0	399.3

Other Rx: Other prescription drugs; OTC: Over-the-counter drugs; Alternatives: Alternative and nonpharmacological treatments. Marginal utilities are presented with standard errors in parentheses. The standard errors are adjusted for clustering on groups of physicians by gender and age

^*^ *p*<0.1, ^**^ *p*<0.05, ^***^ *p*<0.01

## Results

Estimation results from Model 1 and Model 2 are reported in Table 6. The average intervention effect is quantified in Model 1 by assuming the intervention effects on marginal utility of prescribing are fixed over drug types (*γ*_*t*_=*γ*,∀*t*∈{1,2,3,4}). The less restrictive Model 2 allows for heterogeneous intervention effects on marginal utilities for the four types of drugs.

In panel A in Table 6, we present the estimates of the mean marginal utilities for each of the four drugs without the intervention, *v*_*t*_, with robust standard errors in parentheses. In panel B we report the average intervention effect (*γ* in Model 1), while heterogeneous intervention effects (*γ*_*t*_ in Model 2) are reported in panel C. For both models, the mean marginal utility of prescribing without the intervention (panel A) differs substantially over the four drug types. The mean marginal utilities are positive for Antibiotics and OTC, and negative for Other Rx and Alternatives. Negative mean marginal utilities are expected for Other Rx and Alternatives, as only a minority of physicians included these types of drugs when treating a pseudopatient. In panel B for Model 1, we see that the estimated average intervention effect is negative and statistically significant. The interpretation is that the mystery shopper intervention caused a reduction in the mean marginal utility, and thus reduced the probability of prescribing drugs to the pseudopatient.^8^

An important aspect of homogeneous effect models like Model 1 is that they may conceal systematic intervention effects in cases where the intervention increases prescribing of some drugs and reduces that of other drugs, implying that the intervention causes behavioral changes. The fact that substitution is a rational response by an economic agent is in general an important issue to consider when conducting experiments in the field [43]. In Model 2, we account for the possibility of substitution by allowing for between-drug-variation in the intervention effect. The heterogeneous intervention effects are presented in panel C. The Akaike information criterion (AIC) and the Bayesian information criterion (BIC) do not indicate substantial differences in fit when comparing the two models. However, the hypothesis that the intervention effect, *γ*_*t*_, is independent of drug type can be rejected (*p*−*value*=0.0029, Wald test). The interpretation of the heterogeneous intervention effects is that the announcement of a mystery shopper audit led to a reduction in prescribing of Other Rx and OTC.^9^

While the conditional logit model provides consistent estimates of the mean marginal utilities and intervention effects, heterogeneity in these parameters are not modeled explicitly. To provide inference on differences in means, while allowing for the possibility of heterogeneous effects, we apply cluster-robust standard errors [44].^10^ We estimate the cluster-robust standard errors by grouping physicians according to their gender and age,^11^ and a summary of the clustered groups is presented in Table 7. We describe the robustness to alternative criteria for clustering physicians in Appendix B.

**Table 7:** Summary of groups

	**Young female**	**Young male**	**Old female**	**Old male**	**Total**
Control	13	5	15	14	47
Treatment	13	15	9	10	47
Total	26	20	24	24	94

Notes: Physicians older than 40 years old are grouped as “Old”, and those younger than 40 are grouped as “Young”

To enhance the credibility of the research project in the intervention, we offered the clinics in the treatment group options for receiving feedback of the quality assessment. The three options were: publicly available feedback (results will be published on the Shandong University website), feedback in private (results will only be received by the clinic) or no feedback. Table 8 summarizes physician prescribing behavior by their feedback choices.

**Table 8:** Prescribing behavior and feedback choices

	**No feedback**				**Private feedback**				**Public feedback**			
	**Mean**	**Sd.**	**Freq.**	***N***	**Mean**	**Sd.**	**Freq.**	***N***	**Mean**	**Sd.**	**Freq.**	***N***
Antibiotics	72.7%	0.452	24	33	45.5%	0.522	5	11	100%	0	3	3
Other Rx	12.1%	0.331	4	33	0	0	0	11	33.3%	0.577	1	3
OTC	78.8%	0.415	26	33	72.7%	0.467	8	11	66.7%	0.577	2	3
Alternatives	0	0	0	33	18.2%	0.405	2	11	33.3%	0.577	1	3

Among all 47 physicians, 33 chose to receive no feedback, 11 opted into receive private feedback, while only 3 were willing to publish their evaluation results on the University website. It is worth mentioning that providing physicians with feedback options was not designed to reveal any causal relation between feedback choice and prescribing behavior. The reason is that feedback choices made by the physicians are endogenously decided, not exogenously assigned to clinics. Nevertheless, we report the prescribing behavior of four types of drugs in three feedback groups below and encourage future study designs on the relationships between feedback choices and prescribing behavior.

## Discussion

Overprescribing of medications contributes to rising health expenditures and possibly adverse health outcomes. Unlike many previous studies, which have focused only on the overprescribing of antibiotics, we investigated the intervention effects on four types of drugs, including antibiotics, other prescription drugs, over-the-counter drugs, and alternative and nonpharmacological treatments. We quantified the change in composition of prescriptions caused by the intervention. Our results provide evidence that there is substantial variation in prescribing in the case of a mild common cold. Moreover, we found that the average intervention effect is mostly driven by reductions in Other Rx and OTC medications.

The finding that an announcement of a mystery shopper audit does not have significant effect on antibiotic prescribing might have several explanations: The intervention message did not provide any specific assessment criteria on the quality of primary care, and thus physicians’ response to the intervention might reflect their prioritization of good quality. Furthermore, prescribing medications that satisfy the patients’ expectations might be one of the quality aspects that is considered important to clinics for attracting patients. Due to the limited awareness of antibiotic resistance and lack of knowledge on antibiotic misuse in the population, patients demand antibiotics for self-medication [45–48], and expect primary care physicians to provide antibiotics [25, 49]. Antibiotics are often prescribed due to diagnostic uncertainty as it is difficult to distinguish whether an infection is viral or bacterial, especially at the early stage [50–52].

The credibility of the pseudopatients is a key issue, and, in particular, it is important that the physicians were not able to identify them. It is important to note that the script for the symptom presentation from Currie et al. [26, 27] is deliberately developed so that the physicians cannot observe from an examination whether or not the pseudopatient’s presentation is true. The pseudopatient’s presentation cannot be proven false objectively. While the announcement of audits might make physicians alert for pseudopatients, vague symptoms of the common cold are so prevalent among patients in general that it is hardly feasible for physicians to dismiss this type of patient. There are obviously other patients who have symptoms that can easily be verified, hence, physicians might feel confident that those patients are not the mystery shoppers. Therefore, our effect estimates should be interpreted in the context of the common cold where the issue of overprescribing is highly relevant.

One might be concerned about information spillover among individual physicians from different groups. Since the intervention was randomly assigned to the clinics, we could not control for the distance between clinics in the treatment and control groups. Even though we were informed that there was no association or organized union of primary care clinics in Jinan where physicians could exchange information on a regular basis, we cannot rule out the possibility of information spillover about the intervention among individual physicians from different groups. Given our experimental design, however, we expect information spillover to have a minor impact, if present at all. If information about the intervention reached the clinics in the control group, they would most likely expect a mystery shopper audit to be preceded by an announcement. Hence, one reasonable strategy for a clinic in the control group is to not change behavior. In the case where a clinic in the control group does change behavior and reduce prescribing, it would result in a smaller intervention effect compared to a situation where information spillover is absent.

Field experiments cannot facilitate a perfectly controlled environment. The behavior of individuals in the treatment group might affect that in the control group in some indirect way which is unobservable to the researchers. While the pseudopatients’ behaviors in our experiment were predetermined and therefore unaffected by physician behavior, one can never completely rule out the possibility of behavioral spillovers when conducting experiments in the field.

Our study investigated the intervention effect three weeks after the intervention. More research is needed in order to provide knowledge on the long-term effects of a mystery shopper scheme.

## Conclusion

In health care systems where provider performance data and patient registers are not available, interventions that can be implemented to influence asymmetric information and thus improve health care quality are of great interest to policy makers. This study provides new evidence suggesting that announced performance auditing of primary care providers could directly affect physician behavior, even when it is not combined with pay-for-performance or measures such as reminders, feedback or educational interventions. In our study, we conducted a field experiment to assess the impact of a preannounced mystery shopper audit on prescribing behavior in primary care in China. We find that the mystery shopper intervention reduces the probability of prescribing. Moreover, we find that the intervention effects are heterogeneous and differ across types of medicine. We present robust evidence suggesting that a simple announcement of a mystery shopper scheme influences medical treatment decisions. Hence, our results suggest that, upon making medical decisions, physicians have a rich set of motives that do not only include profit and health benefits. More knowledge regarding these motives is needed to develop policies that improve welfare.

## Appendix

### A First audit

In this section, we show the balance of the randomization by analyzing the ”intervention effect” on prescribing behavior in the first audit. The first audit was conducted one week before the intervention, and 96 clinics were randomly grouped into control and treatment. We expect that the assignment of groups does not affect physicians’ prescribing behavior. The analytical models used here are identical to those for the second audit analyses. Table 9 below reports the results in terms of marginal utilities. Not surprisingly, no intervention effect was detected in the first audit. In addition to the balance of randomization at clinic level which we demonstrated in “Data” section, the results here reinforce the balance at the individual level, providing evidence that physicians in the treatment and control group did not behave significantly differently prior to the intervention. The standard errors are adjusted for clustering on matched groups of physicians by gender and age. Table 10 summarizes the matched groups.

**Table 9:** Intervention effects on physician prescribing, audit 1

	**Model 1**	**Model 2**
**Panel A: Prescribing pattern**		
Antibiotics	0.580^*^	0.511
	(0.306)	(0.264)
Other Rx	-1.967^***^	-1.946^***^
	(0.450)	(0.356)
OTC	1.589^***^	1.768^***^
	(0.304)	(0.499)
Alternatives	-3.455^***^	-3.850^***^
	(0.376)	(0.914)
**Panel B: Average intervention effect**		
	0.041	
	(0.101)	
**Panel C: Heterogeneous intervention effects**		
Antibiotics		0.182
		(0.336)
Other Rx		0.000
		(0.267)
OTC		-0.301
		(0.407)
Alternatives		0.715
		(1.480)
Number of observations	768	768
Log-Likelihood	-155.2	-154.8
Pseudo *R*^2^	0.417	0.419
AIC	320.3	325.5
BIC	343.5	362.7

Other Rx: Other prescription drugs; OTC: Over-the-counter drugs; Alternatives: Alternative and nonpharmacological treatments. Marginal utilities are presented with standard errors in parentheses. In both models, the standard errors are adjusted for clustering on groups of physicians by gender and age

^*^ (*p*<0.1), ^**^ (*p*<0.05), ^***^ (*p*<0.01)

**Table 10:** Summary of matched groups, audit 1

	**Young female**	**Young male**	**Old female**	**Old male**	**Total**
Control	16	10	8	14	48
Treatment	16	12	9	11	48
Total	32	22	17	25	96

Notes: Physicians older than 40 years old are grouped as “Old”, and those younger than 40 are grouped as “Young”

### B Robustness of average intervention effect

Now we check the robustness of the average intervention effect to different criteria of clustering levels on which the standard errors are adjusted for. The physicians were grouped according to their gender (male or female) and age (young or old). In “Results” section, We grouped physicians who were older than 40 years old as “Old”, and those who were younger than 40 as “Young” (referred to as clustering 2 in Table 11 below). This method of grouping reflects the reality in China that physicians in general start their careers in their early 20s and retire around age 60. Moreover, this method provides relatively balanced group sizes.

**Table 11:** Summary of groups, clustering 2

	**Young female**	**Young male**	**Old female**	**Old male**	**Total**
**Panel A: Audit 1**					
Control	16	10	8	14	48
Treatment	16	12	9	11	48
Total	32	22	17	25	96
**Panel A: Audit 2**					
Control	13	5	15	14	47
Treatment	13	15	9	10	47
Total	26	20	24	24	94

Notes: Physicians older than 40 years old are grouped as “Old”, and those younger than 40 are grouped as “Young”

Table 12 and Table 13 show the summaries of clustering 1 and clustering 3 where we define “old” and “young” using threshold age 30 and age 50, respectively. Table 14, clustering 4, presents the summary of clustering by all the combinations of age and gender. In all tables, we report the number of physicians in each group.

**Table 12:** Summary of groups, clustering 1

	**Young female**	**Young male**	**Old female**	**Old male**	**Total**
**Panel A: Audit 1**					
Control	0	2	24	22	48
Treatment	2	0	23	23	48
Total	2	2	47	45	96
**Panel A: Audit 2**					
Control	2	0	26	19	47
Treatment	0	2	22	23	47
Total	2	2	48	42	94

Notes: Physicians older than 30 years old are grouped as “Old”, and those younger than 30 are grouped as “Young”

**Table 13:** Summary of groups, clustering 3

	**Young female**	**Young male**	**Old female**	**Old male**	**Total**
**Panel A: Audit 1**					
Control	20	18	4	6	48
Treatment	25	21	0	2	48
Total	45	39	4	8	96
**Panel A: Audit 2**					
Control	25	14	3	5	47
Treatment	21	19	1	6	47
Total	46	33	4	11	94

Notes: Physicians older than 50 years old are grouped as “Old”, and those younger than 50 are grouped as “Young”

**Table 14:** Summary of groups, clustering 4

	**Female**	**Male**	**Female**	**Male**	**Female**	**Male**	**Female**	**Male**	**Total**
	**≤30**	**≤30**	**[31,40]**	**[31,40]**	**[41,50]**	**[41,50]**	**≥51**	**≥51**	**Total**
**Panel A: Audit 1**									
Control	0	2	16	8	4	8	4	6	48
Treatment	2	0	14	12	9	9	0	2	48
Total	2	2	30	20	13	17	4	8	96
**Panel A: Audit 2**									
Control	2	0	11	5	12	9	3	5	47
Treatment	0	2	13	13	8	4	1	6	47
Total	2	2	24	18	20	13	4	11	94

Notes: Age is categorized into four levels: younger than or equal to 30 years old; between 31 to 40 years old; between 41 to 50 years old; older than or equal to 51 years old

Applying the same analytical model presented in “Results” section, we tested the robustness of the average intervention effect to each clustering criteria. As it shows in Table 15, no significant intervention effect was detected in the first audit, while in Table 16, the intervention resulted in a significant reduction of mean marginal utility of prescribing. The estimates of the average intervention effects and their significance are consistent across four clustering strategies.

**Table 15:** Robustness of average intervention effects on physician prescribing, audit 1

	**clustering 1**	**clustering 2**	**clustering 3**	**clustering 4**
**Panel A: Prescribing pattern**				
Antibiotics	0.580^*^	0.580^*^	0.580^***^	0.580^*^
	(0.313)	(0.306)	(0.216)	(0.309)
Other Rx	-1.967^***^	-1.967^***^	-1.967^***^	-1.967^***^
	(0.485)	(0.450)	(0.596)	(0.446)
OTC	1.589^***^	1.589^***^	1.589^***^	1.589^***^
	(0.440)	(0.304)	(0.341)	(0.292)
Alternatives	-3.455^***^	-3.455^***^	-3.455^***^	-3.455^***^
	(0.302)	(0.376)	(0.378)	(0.441)
**Panel B: Average intervention effects**				
Average intervention effect	0.041	1.073	0.976	0.939
	(0.070)	(0.101)	(0.232)	(0.231)
Number of observations	768	768	768	768
Log-Likelihood	-155.2	-155.2	-155.2	-155.2
Pseudo *R*^2^	0.417	0.417	0.417	0.417

Other Rx: Other prescription drugs; OTC: Over-the-counter drugs; Alternatives: Alternative and nonpharmacological treatments. Estimated odds ratios are presented with standard errors in parentheses. The standard errors are adjusted for clustering on groups following four clustering criteria.

^*^ (*p*<0.1), ^**^ (*p*<0.05), ^***^ (*p*<0.01)

**Table 16:** Robustness of average intervention effects on physician prescribing, audit 2

	**clustering 1**	**clustering 2**	**clustering 3**	**clustering 4**
**Panel A: Prescribing pattern**				
Antibiotics	0.630^***^	0.630^***^	0.630^***^	0.630^***^
	(0.069)	(0.197)	(0.151)	(0.180)
Other Rx	-1.481^***^	-1.481^***^	-1.481^***^	-1.481^***^
	(0.117)	(0.294)	(0.132)	(0.277)
OTC	1.551^***^	1.551^***^	1.551^***^	1.551^***^
	(0.080)	(0.269)	(0.117)	(0.295)
Alternatives	-2.418^***^	-2.418^***^	-2.418^***^	-2.418^***^
	(0.353)	(0.531)	(0.350)	(0.466)
**Panel B: Average intervention effects**				
Average intervention effect	-0.214^**^	-0.214^***^	-0.214^**^	-0.214^**^
	(0.086)	(0.077)	(0.091)	(0.085)
Number of observations	752	752	752	752
Log-Likelihood	-175.4	-175.4	-175.4	-175.4
Pseudo *R*^2^	0.327	0.327	0.327	0.327

Other Rx: Other prescription drugs; OTC: Over-the-counter drugs; Alternatives: Alternative and nonpharmacological treatments. Estimated odds ratios are presented with standard errors in parentheses. The standard errors are adjusted for clustering on groups following four clustering criteria. Clustering 2 is applied in the models in the main paper

^*^ *p*<0.1, ^**^ *p*<0.05, ^***^ *p*<0.01

### C Scripts of pseudopatient used in first and second audit

**Step one: Statement of the Chief Complaint**

Patient: Hello, doctor. For the last two days, I’ve been feeling fatigued. I have been having a low grade fever, slight dizziness, a sore throat, and a poor appetite. This morning, the symptoms worsened so I took my body temperature. It was 37 ^∘^C.

If pseudo patients are asked questions about symptoms mentioned in the chief complaint, they are supposed to answer appropriately. If the doctor asks about other symptoms not in the chief complaint, then they should say that there are no such symptoms. Answer NO if asked the following questions: Do you feel nauseous? Do you have any phlegm? Do you have any muscle soreness? Have you eaten anything bad or unclean recently? Are you currently taking any medications? Do you have medication at home?

**Step two: Physical Examination** Physician: I’ll give you a physical examination/I will now conduct a physical exam. Physical Examination.

**Step three: Physician’s Diagnoses and Explanation of Findings** Physician: I’ll prescribe [...] for you. If the doctor wants to give you medication, ask what medication it is. Patient: what kind of medication it is? Patient takes a look at the medication and memorizes the name and the pharmaceutical company of the medication.

**Fig. 3:**
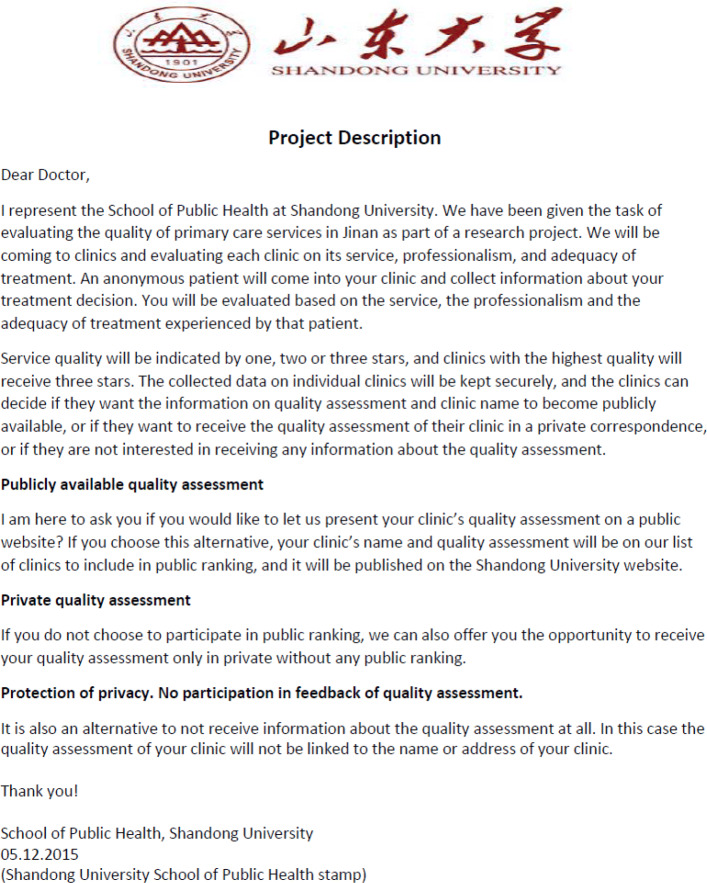
English translation of the project description issued by School of Public Health, Shandong University

**Fig. 4:**
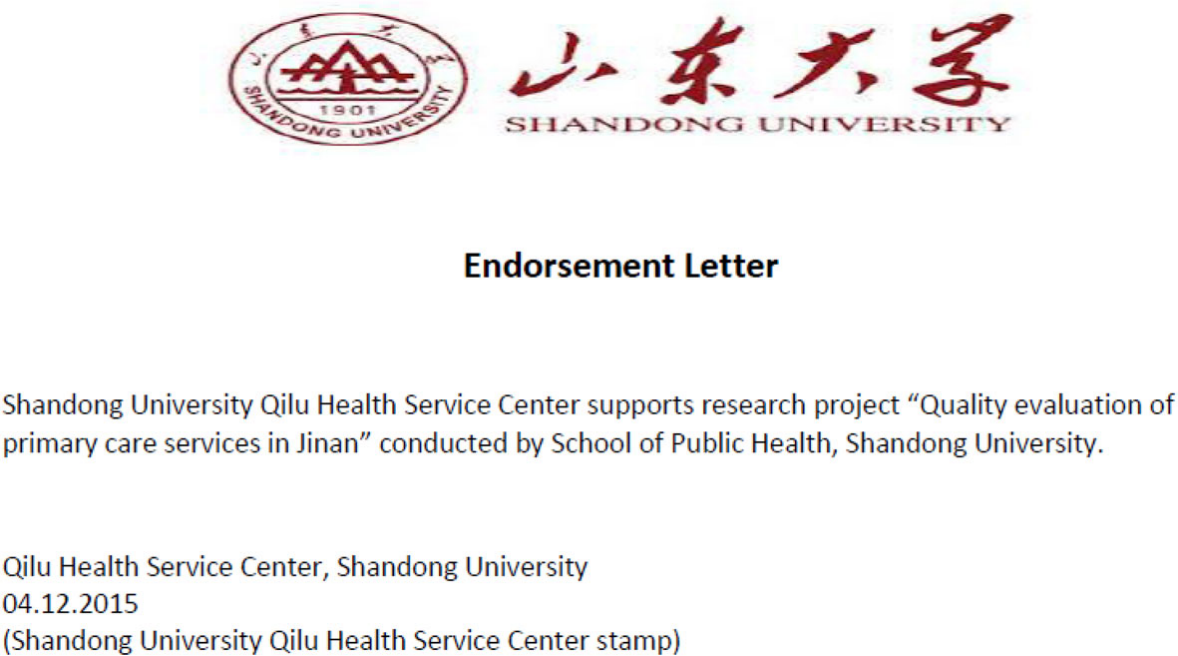
English translation of the endorsement letter issued by Qilu Health Service Center, Shandong University

Ask the physician for information regarding side effects of the medication after 3-4 seconds if the physician does not voluntarily inform you of the side effects. Patient: Ok. [...](pause for 3-4 seconds)[...] Does it have any side effects? If the total is under 20 yuan, buy the medication. Patient: How much is each medication? If it is over 20 yuan, say, Patient: Doctor, I do not have enough money with me today, I can come back later to buy.

**Step four: Departure** Patient: Thank you! Physician: You are welcome.

### D Experimental protocol for the pseudopatient and accompanying student

**Pseudo patient** Before entering the clinic

1. Ensure that you have the questionnaire and IDs are correct.
2. Notify in the chat group that you have arrived at the clinic: WRITE Group XXX arrive at Clinic YYYY.

In the clinic

1. DO NOT say to the doctor that you have a cold.
2. MUST say that you had a slight fever.

Out of the Clinic

1. The two of you fill out the data collection sheet.

**Accompanying student** In the clinic

1. Observe the number of additional patients in the waiting room.
2. Observe the number of additional physicians and patients in the office, the gender and age of the practicing physician.
3. Memorize the name(s) of the medication and the pharmaceutical company.

Out of the Clinic

1. The two of you fill out data collection sheet.

### E Letters used in the intervention

The project description letter was issued by School of Public Health, Shandong University.

The endorsement letter was issued by Qilu Health Service Center, Shandong University.

## Data Availability

All data generated or analysed during this study are included in this published article and its supplementary information files.

## Abbreviations

QOF: Quality and outcomes framework
Rx: Prescription drugs
OTC: Over-the-counter drugs
Alternatives: Alternative and nonpharmacological treatments
MWW: Mann-whitney-wilcoxon
AIC: Akaike information criterion
BIC: Bayesian information criterion

## Footnotes

1 Case number: 44243.

2 There is no third party payer in our study.

3 Other districts or counties are too far away.

4 We excluded dentistry and clinics providing only Chinese medicine because they do not suit our scripted audit scenario.

5 After the experiment, feedback was indeed provided to those physicians who had opted in. The intention of providing physicians with feedback options is to enhance the credibility of the intervention, not to attempt to draw any causal relation between feedback choice and prescribing behavior. The reason is that feedback choices made by the physicians are endogenously decided, not exogenously assigned to clinics.

6 In total, 13 pseudopatients and 13 accompanying students were recruited, allowing for one pair of students to serve as a backup pair.

7 It is not uncommon in China that patients wait in the physician’s office, especially for small clinics.

8 Compared to the control group, the mystery shopper intervention reduced the odds of prescribing by 19.2% as measured by the average intervention effect.

9 Compared to the control group, the mystery shopper intervention reduced the odds of prescribing Other Rx by 61.0%, and reduced the odds of prescribing OTC by 42.7%.

10 One might argue that applying a mixed logit model instead would be preferable, since it would account for the heterogeneity of preferences. The estimation of a mixed logit model with random coefficients would double the number of unknown parameters to estimate, and hence require either a larger number of decision-makers or a larger number of choice occasions for each decision-maker to provide sufficient statistical power.

11 We group physicians who are older than 40 years old as “Old”, and those who are younger than 40 as “Young”. This method of grouping reflects the reality in China that physicians in general start their careers in their early 20s and retire around age 60. Moreover, this method provides relatively balanced group sizes. See Appendix B for a detailed description of other clustering criteria and the robustness check of the average intervention effect.
